# The Pros and Cons of the Use of Laser Ablation Synthesis for the Production of Silver Nano-Antimicrobials

**DOI:** 10.3390/antibiotics7030067

**Published:** 2018-07-28

**Authors:** Maria Chiara Sportelli, Margherita Izzi, Annalisa Volpe, Maurizio Clemente, Rosaria Anna Picca, Antonio Ancona, Pietro Mario Lugarà, Gerardo Palazzo, Nicola Cioffi

**Affiliations:** 1 Dipartimento di Chimica, Università degli Studi di Bari “Aldo Moro”, via E. Orabona 4, 70126 Bari, Italy; maria.sportelli@uniba.it (M.C.S.); m.izzi@studenti.uniba.it (M.I.); m.clemente8@studenti.uniba.it (M.C.); rosaria.picca@uniba.it (R.A.P.); gerardo.palazzo@uniba.it (G.P.); 2 Institute of Photonics and nanotechnology-National Research Council (IFN-CNR), Physics Department “M. Merlin”, Bari, Italy, via Amendola 173, 70126 Bari, Italy; annalisa.volpe@ifn.cnr.it (A.V.); pietromario.lugara@uniba.it (P.M.L.)

**Keywords:** silver nanoparticles, laser ablation synthesis in solution, nano-antimicrobials, food packaging

## Abstract

Silver nanoparticles (AgNPs) are well-known for their antimicrobial effects and several groups are proposing them as active agents to fight antimicrobial resistance. A wide variety of methods is available for nanoparticle synthesis, affording a broad spectrum of chemical and physical properties. In this work, we report on AgNPs produced by laser ablation synthesis in solution (LASiS), discussing the major features of this approach. Laser ablation synthesis is one of the best candidates, as compared to wet-chemical syntheses, for preparing Ag nano-antimicrobials. In fact, this method allows the preparation of stable Ag colloids in pure solvents without using either capping and stabilizing agents or reductants. LASiS produces AgNPs, which can be more suitable for medical and food-related applications where it is important to use non-toxic chemicals and materials for humans. In addition, laser ablation allows for achieving nanoparticles with different properties according to experimental laser parameters, thus influencing antibacterial mechanisms. However, the concentration obtained by laser-generated AgNP colloids is often low, and it is hard to implement them on an industrial scale. To obtain interesting concentrations for final applications, it is necessary to exploit high-energy lasers, which are quite expensive. In this review, we discuss the pros and cons of the use of laser ablation synthesis for the production of Ag antimicrobial colloids, taking into account applications in the food packaging field.

## 1. Introduction

Due to their unique properties [1], metal nanoparticles (NPs) have been used for applications in several fields, such as medicine and the biomedical sciences [2,3], cosmetics [4,5], food and agriculture [6,7,8,9], electronics [10], energy science [11], and catalysis [12], providing significant improvements in each area. This review focuses on laser ablation synthesis in solution (LASiS) nanotechnology and its specific potentialities in the food industry, with particular consideration to food packaging.

The growing demand for ready-to-eat food products, along with requirement of easy and safe transport, has led to the need to extend their shelf life, prevent foodborne diseases, minimize industrial processing, track them, and improve their preservation during storage. To this aim, the use of antimicrobial metal nanoparticles is continuously increasing. They are used in agriculture (e.g., pesticide and fertilizer delivery [13,14,15,16]), food processing (e.g., encapsulation of flavor or odor enhancers, food textural or quality improvement), food packaging (e.g., limitation of pathogen proliferation, gas sensors; UV protection, more impermeable polymer films), and nutrient supplements (e.g., nutraceuticals with higher stability and bioavailability) [17,18,19]. 

Inorganic or organic nanoparticles can either be placed on the surface of polymeric matrices used for food packaging or dispersed into their bulk [20]. The introduction of nanoparticles in a polymeric matrix aims to improve the properties of traditional packaging, e.g., containment and protection (ease of transportation and avoided leakage or break-up), foodstuffs preservation (protection against microbial contaminants, extended shelf-life), convenience (consumer-friendly products), and marketing and communication (real-time information about the quality of enclosed foodstuffs) [8]. In response to required features, food packaging employs innovative materials, which can be categorized as follows:

(1) Improved nanomaterials (the presence of nanoparticles in the polymeric matrix improves the mechanical and/or chemical properties of the packaging, but they are not in direct interaction with food);
(2) Active nanomaterials (dispersed nanoparticles into polymeric bulk enable the packaging to interact actively with environment and regulate the preservation of food);
(3) Intelligent nanomaterials (packaging is able to monitor and identify the state of the product, because of the integration of nanosensors and devices) [8,21].

Nanoparticles, and specifically silver nanoparticles (AgNPs), can be widely used for active packaging due to their antimicrobial properties [22]. Generally, organic antimicrobial materials are less stable at high temperatures compared to inorganic ones, whereas metal and metal oxide nanoparticles withstand harsher processing conditions [23].

The most common nanocomposites used as antimicrobial films for food packaging are based on AgNPs, which are well-known for their efficacy towards a wide range of microorganisms, with high temperature stability and low volatility [20].

Several reviews pick features and use common nanostructures employed in food packaging [8,20,21,24,25,26,27,28,29,30,31]. In each of them, AgNPs are taken into account, but few papers are exclusively focused on AgNPs in food packaging [23]. One of the earliest works on this specific topic was proposed by Rhim and coworkers; they produced chitosan–AgNP nanocomposite films and examined their bioactivity and mechanical properties [32]. 

In general, AgNP-based nanocomposites are stable and offer slow release of silver ions in the surrounding medium, resulting in a long-lasting antimicrobial activity [8]. The released amount of silver ions into the system is dependent not only on the properties of the nanoparticles themselves—for example NP size, shape, structure, composition, etc.—but also relies on external factors, including the properties of the surrounding medium: ionic strength, pH, composition, humidity, dissolved oxygen content, temperature, etc. [26].

This review is focused on AgNPs to be used in food packaging, and particularly on a peculiar NP synthesis approach. Special attention will be given to AgNPs synthetized by laser ablation, a method proposed by several groups as a green route to high-purity nanomaterials. The pros and cons of this technique for the production of Ag antimicrobial nanocolloids will be critically discussed.

## 2. Silver Nanoparticles

AgNPs are chemically stable [33,34] antimicrobial agents [35] providing strong activity towards a wide range of pathogenic microorganisms, including bacteria, yeasts, viruses, fungi, and parasites, even when low doses are used (full growth inhibition of bacteria can occur at a few mg/mL) [36]. Moreover, AgNPs are non-toxic to the human body at low concentrations [37]. The Occupational Safety and Health Administration (OSHA) and Mine Safety and Health Administration (MSHA) proposed that a permissible exposure limit (PEL) for metallic and most soluble Ag compounds should be 0.01 mg/m^3^. Argentina, Bulgaria, Columbia, Jordan, Korea, New Zealand, Singapore, and Vietnam recognize the American Conference of Governmental Industrial Hygienists (ACGIH) threshold limit values (TLV) of 0.1 mg/m^3^ for metallic Ag, while Austria, Denmark, Germany, the Netherlands, Norway, Switzerland, and Japan recognize 0.01 mg/m^3^ as the occupational exposure limit for all forms [38]. According to the Registration, Evaluation and Authorization of Chemicals (REACH; Council of the European Union for chemicals and nanomaterials regulation), 0.01 ppb of Ag (for medical products) is not an environmental concern, even if this threshold cannot be interpreted as a safe concentration [39]. The European Food Safety Authority (EFSA) Panel on Food Additives and Nutrient Sources Added to Food did provide upper limits of Ag migration from packaging. Recommended values should not exceed 0.05 mg/L in water and 0.05 mg/kg in food. This implies that the evaluation of silver migration profiles is needed to assure antimicrobial effectiveness while complying with the current legislation, and that products for food packaging and food supplements containing AgNPs are not allowed in the EU, unless authorized [23]. Toxicity of AgNPs also depends on their size, as it generally increases upon decreasing size [40,41]. A smaller size results in the following characteristics: (1) greater tendency to enter into organisms; (2) a larger number of surface atoms available for diverse reactions; (3) more released Ag ions from the nanoparticles; and (4) more reactive oxygen species (ROS) production on the surface, which eventually results in an increased toxicity [40]. However, there are many papers that discuss in detail the properties and aspects connected to AgNP. toxicity, such as [38,42,43,44,45,46,47,48,49], to cite a few.

### 2.1. Synthesis Methods 

**Figure 1:**
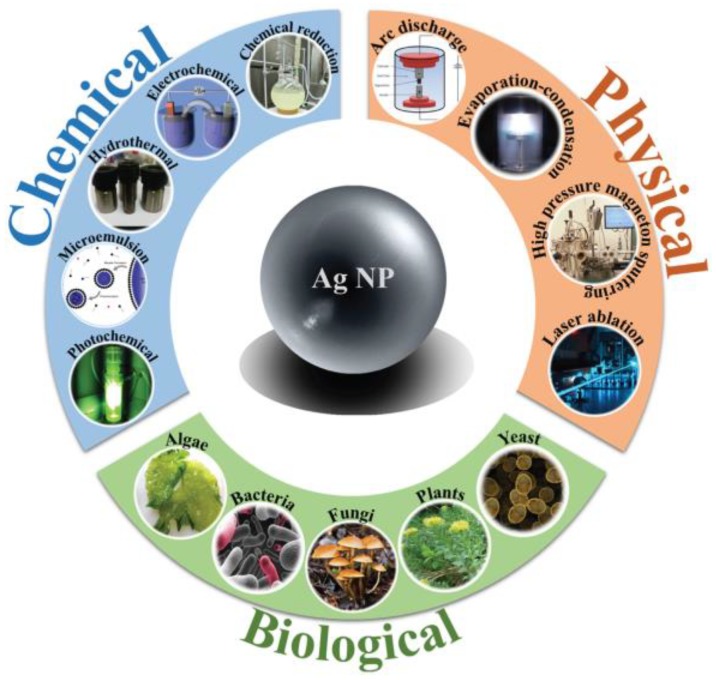
Schematic representation of some of the main methods available for the synthesis of silver nanoparticles (AgNPs). Reprinted from [61], with permission from Elsevier.

The synthesis of supported (which is beyond the scope of this review) and colloidal AgNPs has been investigated extensively and, over the years, several techniques have been proposed for the synthesis of AgNPs. For a detailed view of such literature, we recommend review works and book chapters published in the last two years, such as [50,51,52,53,54,55,56,57,58,59,60,61,62,63,64,65,66], which deal with the synthesis of colloidal materials. Figure 1 summarizes the main approaches to synthetize AgNPs, including chemical reduction [67,68], photoreduction [69,70,71], microemulsion (reverse micelle) methods [72,73], electrochemical methods [74,75,76], evaporation-condensation processes [77], laser ablation [18], and biosynthesis [78,79].

Chemical reduction is the most common method for the preparation of AgNPs as stable colloidal dispersions. This method requires a reductant capable of transforming the silver salt into AgNPs, which is usually also used as a stabilizing or capping agent to ensure stability of colloids. Commonly used reducing agents are ascorbic acid, sodium borohydride, sodium citrate, ferulic acid, poly(ethylene glycol)-block copolymers, and hydrazine compounds [64,80,81,82]. Jokar et al. [83] prepared AgNPs for low-density polyethylene (LDPE) nanocomposites via chemical reduction, using polyethylene glycol (PEG) as a reducing agent, stabilizer, and solvent, with silver nitrate (AgNO_3_) as metal precursor. To improve antibacterial activity of AgNPs, researchers usually use reductants and stabilizing agents which possess additional antibacterial properties. Cao and coworkers, for example, proposed the synthesis of AgNPs using AgNO_3_ as a metal precursor, ascorbic acid as reducing agent, and chitosan as a stabilizer. Chitosan, which exhibits excellent biocompatibility, biodegradability, and antibacterial and antifungal activities, was used to prepare silver nanoparticles in many studies [84,85,86]. Sometimes, chemical reduction may be supported by microwave to achieve a more homogeneous heating process and speed up the reaction rate [87].

Biological synthesis of metal nanoparticles using biological agents such as bacteria, fungi, yeast, plant, and algal extracts is becoming more common due to the necessity to develop simple, cost-effective, and eco-friendly processes. The fundamental mechanism is an ordinary chemical reduction, with the difference that reductants are natural agents. Plants and their parts contain carbohydrates, fats, proteins, nucleic acids, pigments, and several types of secondary metabolites which can act as reducing agents to produce nanoparticles from metal salts without any toxic by-products [88]. Similarly, biomolecules such as enzymes, proteins and bio-surfactants present in microorganisms can serve as reducing agents [64]. The major phytochemicals responsible for reducing silver ions into AgNPs are terpenoids, glycosides, alkaloids, and phenolics (flavonoids, coumarins, ubiquinones, tannins, lignin, etc.) [89]. There are countless organisms which can be used for the synthesis of NPs. Bhoir and coworkers [89] used mint extract in the presence of silver nitrate as metal precursors and polyvinyl alcohol (PVA) as a capping material. Moreover, they demonstrated the use of these nanoparticles in food packaging: incorporating them in chitosan and gelatin blend they obtained improved mechanical and barrier properties for the chitosan–gelatin films, as well as antimicrobial activity for food packaging applications. Terenteva at al. [80] investigated the synthesis of AgNPs under the influence of flavonoids as reductants, and they found that quercetin, dihydroquercetin, rutin, and morin produced AgNPs better than chrysin, naringenin, and naringin. 

Most of the synthetic techniques mentioned above may suffer from some drawbacks. With chemical synthesis, metal precursor, reductant, and stabilizing/capping agents, which are always needed to ensure stable chemical-synthetized colloids, are all present in the synthesis solution. However, they (or their by-products) can be toxic and unsafe for human health. For this reason, adverse products must be separated and removed from the final nanocolloids before their use in antibacterial, biomedical, or catalytic applications.

In general, LASiS is a low environmental impact technique which does not need metal precursors and reductants, and produces colloids of a relatively high purity as compared to chemical methods. In particular, the possibility of fragmenting a metal target without making use of capping and reducing agents intrinsically lowers the risk of contamination of the resulting colloid from unknown chemical agents and provides NPs with unique surface characteristics [90]. In the field of nano-antimicrobials, LASiS-generated NPs are expected to exhibit higher reactivity and antimicrobial effects in comparison to their chemically synthesized homologous NPs, due to the absence of ligands and/or stabilizers on the NP surface [91,92]. 

In addition, LASiS allows in situ conjugation of nanoparticles with biomolecules, which has sometimes proved to be more efficient than the ex situ conjugation required for chemically synthetized nanoparticles [93]. 

Hence, high-purity nanoparticles generated by laser ablation can be considered as a very promising agent for antimicrobial applications, especially in food packaging. 

However, in terms of other preparation methods, laser ablation presents some drawbacks as well. Indeed, it incurs high investment costs because of the high price of laser system; to be economically convenient, a large number of colloids should be prepared, and fairly often. Moreover, lasers need a considerable amount of energy (although many other synthesis routes need a high energy consumption) [94], and the most diffused laser sources are not capable of producing nanomaterials on an industrial scale. A great amount of energy is necessary to deliver a good ablation efficiency. Ablation efficiency decreases with long ablation time because of significant number of NPs placed along the laser beam. This inconvenience can be solved through a careful choice of fluidics, e.g., by removing the as-prepared NPs from the optical path by using a flow-through system [95].

Barcikowski and Gökce et al. have recently demonstrated the production of nanocolloids at continuous multi-gram ablation rates (i.e., up to 4 g/h) for different metals and under tight composition control. They exploited a 500 W picosecond laser source working at 10 MHz repetition rate fully synchronized with a polygon scanner in order to reach a scanning speed up to 500 m/s [96,97]. Such a technical solution allows to spatially bypassing the laser-induced cavitation bubbles that prevent higher ablation rates at an MHz repetition rate due to the shielding effect. Therefore, we firmly believe LASiS represents a very interesting and versatile way to produce technologically relevant Ag nano-antimicrobials and in the following we will systematically discuss the aspects which make invaluable this technique. 

### 2.2. Bioactivity of AgNPs 

**Figure 2:**
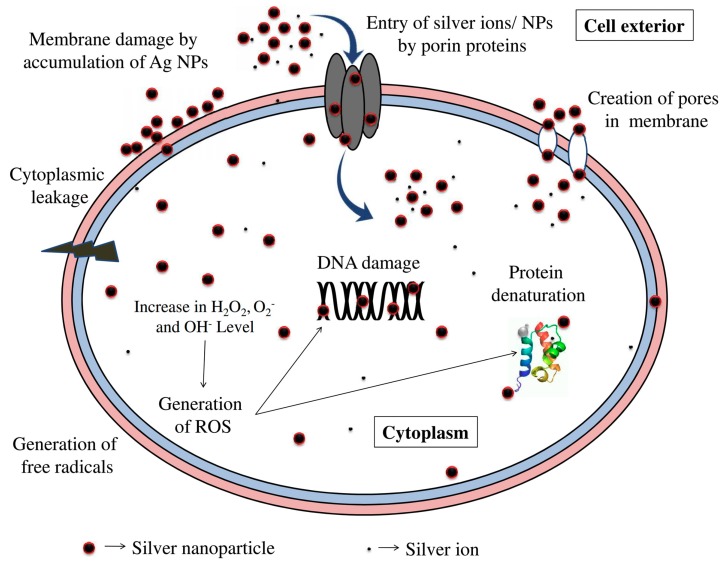
Schematic representation of the known mechanisms of antibacterial action of silver nanoparticles and silver ions. Reprinted from [108], with permission from Elsevier.

Many studies have been performed on the mechanism of action of AgNPs and the complete significance of AgNP bioactivity is still under investigation. A bird’s-eye view of some relevant review works published in the last three years is provided in [36,52,66,98,99,100,101,102,103,104,105,106,107,108,109,110,111,112,113]. A variety of mechanisms may be involved in the antimicrobial activity of silver against a broad spectrum of organisms (Figure 2). Some of the commonly accepted mechanisms include silver–amino acid and silver–thiolate group interactions, silver–DNA interactions, generation of reactive oxidative species, and direct cell membrane damage [47,114].

Zhang and his collaborators highlight that Ag^+^ is expected to show high affinity to the soft base-like thiolate ligands, which are abundant in the bacterial membrane and subcellular structure (e.g., sulfur-containing proteins and enzymes), leading to the inhibition of crucial biological cellular functions [114]. It was found that intracellularly released Ag^+^ ions interact with thiol groups of antioxidants such as glutathione (GSH), superoxide dismutase (SOD), and thioredoxin, leading to increased lipid peroxidation, oxidative stress, DNA damage, and subsequent apoptotic cell death [100]. Nomiya and coworkers found Ag^+^ bonded to the amino acids, forming weak Ag–N bonds which replace biological bonds, resulting in the alteration of cell machinery [115]. AgNPs in the mitochondria could potentiate mitochondrial membrane potential collapse, disruption of the respiratory chain, oxidative stress, inhibition of ATP synthesis, and subsequent activation of the mitochondria-dependent intrinsic pathway of apoptosis. NPs are able to attach to the bacterial membrane by electrostatic interaction [100,114,116]. Lara et al. reported that the positive charge on the Ag^+^ ion is crucial for its antimicrobial activity through the electrostatic attraction between the negatively-charged cell membrane of the microorganism and the positively-charged inorganic agent. According to these authors, one of the mechanisms of antibacterial action for AgNPs is the formation of pits in the cell wall due to AgNP accumulation in the bacterial membrane, which disturbs membrane permeability, resulting in membrane degradation and cell death [37]. Furthermore, AgNPs and Ag^+^ ions can work as catalysts and increase generation of reactive oxygen species (ROS). ROS are usually present in cells in small amounts, but an excess can lead to oxidative stress [47,117]. Nanosilver is also known to interact with DNA and cause DNA damage [118]. DNA is responsible for the reproduction process. Any damage done to it will cause either mutation or death of the organism. It was found that AgNPs specifically interact with the exocyclic nitrogen present in the adenine, guanine, and cytosine bases, which leads to DNA changes [101]. Undoubtedly, both Ag^+^ ions and AgNPs possess antimicrobial activity, but it is very hard to precisely discriminate between the effect of ions and those of nanosilver [44]. Li et al. [119] reported on a similar mode of action of Ag^+^ ions compared to that of AgNPs, although with stronger antibacterial activity. Navarro et al. [120] suggested that AgNP toxicity could be explained by the release of Ag+ from the particles which damage cells.

**Figure 3:**
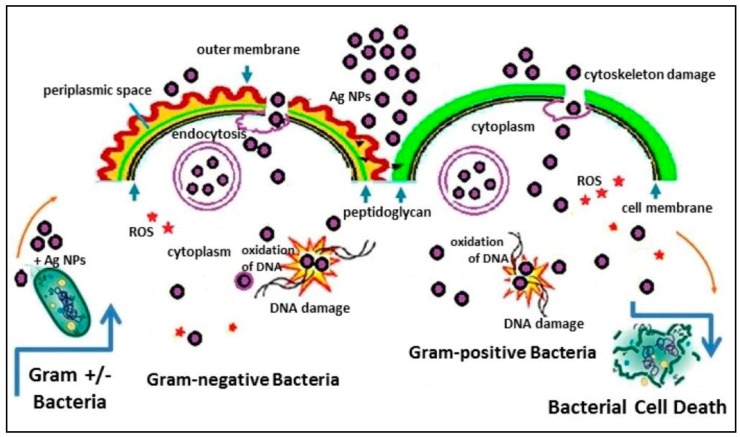
Schematic diagram of bactericidal activity of AgNPs on Gram-positive and Gram-negative bacteria. Reprinted from [105], an open access article distributed under the Creative Commons Attribution License. ROS: reactive oxygen species.

AgNPs have been proved to be active against both Gram-positive and Gram-negative bacteria, as briefly listed in Table 1, where effect of both silver ions and AgNPs has been described on several microorganisms [103,121]. It is well accepted that AgNPs and Ag^+^ give rise to different cellular uptake pathways (Figure 3) depending on whether Gram-positive or Gram-negative bacteria are considered, as also shown in some recent papers [103,122]. 

AgNPs have also been proved to be active against multi-resistant bacteria like methicillin-resistant *Staphylococcus aureus* (MRSA), as well as multidrug-resistant *Pseudomonas aeruginosa*, ampicillin-resistant *Escherichia coli O157:H7* and erythromycin-resistant *Streptococcus pyogenes* [37], or other pathogenic organisms such as *Bacillus subtilis*, *Vibrio cholera*, and *Syphilis typhus* [64]. Pazos-Ortiz and colleagues showed antibacterial activity of AgNPs dispersed in polycaprolactone (PCL). They found that there is greater sensitivity towards *Escherichia coli*, *Klebsiella pneumoniae*, *Staphylococcus aureus*, and *Pseudomonas aeruginosa*, and poor results for *Bacillus subtilis* and *Streptococcus mutans* [123]. Gram-negative bacteria (e.g., *Escherichia coli*, *Pseudomonas aeruginosa*, and *Klebsiella pneumoniae*) were found to be, in general, more sensitive to AgNPs than Gram-positive ones (e.g., *Staphylococcus aureus*, *Streptococcus mutans*, and *Bacillus subtilis*), because of their negatively charged external membrane together with the thinner peptidoglycan layer, which allows adherence and the subsequent penetration of AgNPs [26,123].

**Figure 4:**
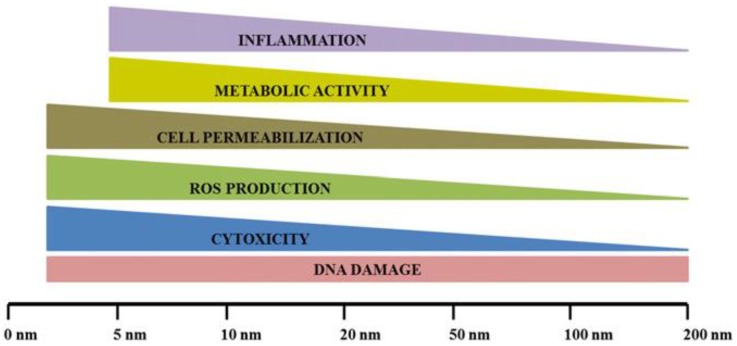
Size-dependent effects of AgNPs in vitro. In general, adverse cellular effects are associated with exposure to smaller AgNPs. One exception is DNA damage; the magnitude of response appears to not depend on AgNP size. Reprinted from [100], with permission from Elsevier.

The biological activity of AgNPs depends on factors including surface chemistry and morphology, size, shape, coating/capping agent, NP agglomeration and dissolution rate, particle reactivity in solution, and efficiency of ion release [136,137,138,139,140]. According to Riaz Ahmed and colleagues [100], smaller sized AgNPs (about <20 nm) with increased surface to volume ratio possess increased cell permeation capacity and a higher rate of Ag^+^ ion release, thus increasing the potential for cytotoxicity and cell injury. They proposed an empiric scale of AgNP bioactivity depending on size (Figure 4) and they showed that cellular effects aggravate with size decrease. They also outlined how entity of DNA damage is not dependent on AgNP diameter.

It has been observed that the shape of AgNPs also causes a critical impact on their antimicrobial activity. Plate- and rod-shaped AgNPs showed higher antibacterial activity as compared to spherical AgNPs and thus they could be used in lower concentrations. In fact, it was observed that the bactericidal activity of plate- and rod-shaped AgNPs was favored by the presence of high atom density facets {111}, whereas, due to predominance of {100} facets on spherical AgNPs, the latter showed relatively lesser bactericidal activity [108,127]. The concentration of AgNPs is another important factor affecting toxicity. It is critical to determine the minimum concentration level of NPs that induces toxicity and its variation in different subjects. Table 2 summarizes a few works that discussed a concentration-dependent AgNP bioactivity. 

AgNPs produced by laser ablation ensure surface cleanliness and the absence of capping agents, which could induce a potential shielding effect on the antimicrobial activity. Hence, we would expect that the antimicrobial activity of laser-produced nanoparticles would be higher compared with the bioactivity of colloids fabricated with other methods which result in NPs with a core-shell structure. Furthermore, reaction by-products of AgNPs synthesis may have potential toxicity. This poses serious issues for the authorization of use of these nanomaterials. However, there are only a few studies that analyze the bactericidal properties of AgNPs produced by laser ablation, and a systematic assessment of these aspects is still missing.

Perito and coworkers [146] tested the antimicrobial activity of AgNPs prepared either by nanosecond (ns) or picosecond (ps) laser ablation using a 1064-nm ablation wavelength, in pure water and in LiCl solution against two bacteria: *E. coli*, as a model for Gram-negative bacteria, and *B. subtilis*, as a model for Gram-positive bacteria. They found that silver colloids ablated in chloride solution exhibited higher antimicrobial activity compared to colloids ablated in pure water. They suggested that AgNPs are coated by a thin oxide layer which “activates” AgNP surface by the addition of a small quantity of LiCl, increasing metal surface reactivity due to the presence of positively-charged active sites. They also stated that bacterial growth inhibition is more effective with AgNPs having an average diameter lower than 10 nm (i.e., prepared with ns pulses), and this is in agreement with the findings of several other works [129,147,148,149,150]. As a general statement supported by different groups, it can be inferred that the antibacterial activity of AgNPs decreases with increasing particle size [88].

The effect of the AgNP concentration on the final biocidal properties, while intuitive and reasonable, is not obvious in the case of use of silver nano-antimicrobials because of the strict solubility limits that Ag^+^ ions show in vivo, primarily due to the precipitation of insoluble salts such as AgCl, which could lower bioactivity. Nevertheless, Korshed and coworkers studied laser-generated AgNPs [151] and showed that the NP antibacterial effects against both Gram-negative and Gram-positive bacteria displayed significant dose dependency on AgNP concentration when investigating a range from 10 μg/mL to 50 μg/mL. Similar trends were observed by Pandey and colleagues, investigating a concentration range from 40 μg/mL to 600 μg/mL [122].

Zafar et al. [152] reported a comparison between the bioactivity of AgNPs produced by laser ablation and AgNPs produced by chemical reduction. The size of chemically synthesized nanoparticles was in the range of 30–40 nm, while the size range of laser ablated nanoparticles was 20–30 nm. Experiments were carried out at the same AgNP dose and laser-ablated nanoparticles provided maximum inhibition against each pathogen (*S. aureus*, *E. coli*, *Salmonella*). The reduced bioactivity of chemically synthesized NPs, as compared to laser-ablated ones, was interpreted as due to the adsorption of chemical species on their surface, producing adverse effects on their antibacterial action. However, NP size plays a key role in the antibacterial action, so the different bioactivity of laser-ablated AgNPs and chemically synthetized-AgNPs shown in this paper could also be ascribed to different NP sizes. 

Based on the literature cited above, it is evident how both the effect of NP size and concentration strictly depends on the single microorganism involved. As a general interpretation, it is possible to state that, even though <10 nm NPs can perform a stronger antimicrobial action, it is better to maintain a conservative approach towards potential nanotoxicological issues arising from the use of such small materials. It is known, in fact, that risks related to NP penetration through main entrance pathways in the human body reach a maximum for dimensions below a critical value of ~50 nm in diameter [153]. Hence, in a growing number of applications of NP-based materials in real-life products, the presence of a (polymeric) matrix that immobilizes NPs appears to be extremely important. In fact, it can prevent and/or limit human exposure to bare (and potentially dangerous) NPs [154].

## 3. Laser Ablation Synthesis in Solution 

In the laser ablation process, an extremely high energy is concentrated at a specific point on a solid target, to remove the material from surface. When a laser pulse irradiates the surface of a bulk material, electromagnetic radiation is adsorbed by the target electrons and energy transfers to material vibrational lattice. As a result, material is expelled from the surface in the form of a plasma plume (in which nanoparticles are formed) [155]. The plasma plume is confined, due to the high pressure exerted by the surrounding liquid, and the considerable temperature gradient between the plume and the liquid. During the plasma decay, the energy is transferred to the surrounding liquid, producing a layer of vapor with a volume approximately equal to that of the plasma, and shaping up a cavitation bubble. Soon after, the cavitation bubble undergoes a periodic evolution of further expansion and shrinkage until its collapse, after which nanoparticles are released into the environmental liquid [156].

The ablation rate is generally determined by laser parameters such as: wavelength, fluence, pulse duration and repetition rate, light absorption efficiency of the target material, transmission, and chemical composition of the liquid. Consequently, NP features depend on laser parameters as well as on the liquid medium. Typical requirements for laser ablation are a wavelength from UV-Vis to near infrared (NIR-IR), a laser fluence approximately comprised between 0.1 and 100 J/cm^2^, pulse durations from nanosecond (ns) to picosecond (ps) and femtosecond (fs) [92,95].

These laser parameters can be used to tune several NP features, such as size, shape, surface properties, aggregation state, solubility, structure, and chemical composition [156,157]. As we have previously discussed, these features may affect the NP antimicrobial activities: hence, it is important to know how NP characteristics depend on some laser parameters.

### 3.1. Ablation Medium

Distilled or deionized water is the most frequently employed liquid medium for the LASiS synthesis of metal nanoparticles, as shown by Mafuné et al. [19,158,159,160,161]. Using water as synthesis medium could generate several oxide or hydroxide species because of reactions occurring between the target material and dissolved oxygen, or oxidizing reactions caused by the plasma-induced decomposition of water. Species (such as hydroxyl groups) can be adsorbed on the NP surface, which can lead to highly-charged surfaces that contribute to the electrostatic stabilization of the synthetized nanoparticles [156]. Water is a favorable medium in most ablation processes because it is cheap, safe, exhibits a high heat capacity, and does not absorb laser light [162].

Organic solvents have been also investigated for laser ablation processes, and the most commonly used ones are methanol, ethanol, isopropanol, acetonitrile, and ethylene glycol. When using organic solvents, the higher dipole moment of the solvent has been reported to result in a higher ablation efficiency and in smaller particles. This effect was attributed to the increased electrostatic interactions resulting from the higher molecular dipolar moment of the solvent molecules, which generates a stronger electric double layer at the NPs surface and enhances the repulsive force between NPs [156]. Comparing organic solvents with different viscosity and different dipole moment, it was found that the smallest and most stable AgNPs, with the narrowest size distribution, were obtained in acetone and 2-propanol. In fact, the former has high dipolar moment but low viscosity, while the latter has high viscosity and low dipolar moment. Hence, factors like solvent dipolar moment and viscosity play a fundamental role in avoiding NP agglomeration [163]. Furthermore, when using homologous solvents, such as alcohols with different chain lengths, it has been shown that short-chain alcohols (e.g., methanol and ethanol) result in unstable particles, whereas alcohols with chain lengths from C-3 to C-5 give rise to more stable and smaller particles as compared to those produced in alcohols with chain lengths exceeding C-5 [164].

**Figure 5:**
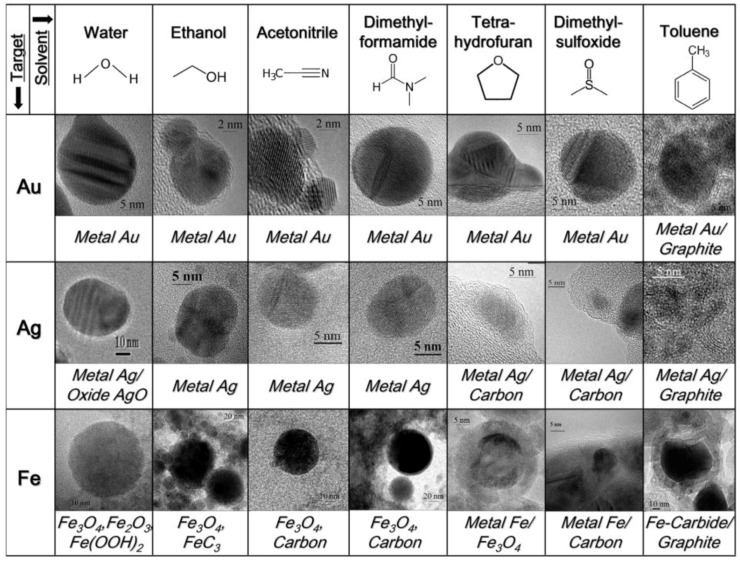
Summary of the NMs obtained by laser ablation of Au, Ag, and Fe bulk targets in different solvents with 9-ns pulses at 1064 nm and 10 J cm^−2^. Reprinted from [166], an open access article distributed under the Creative Commons Attribution License.

Moura et al. [165] showed that ethanol and acetone can be good stabilizing environments to keep NPs free from precipitation and oxidation; however, organic environments resulted in a low process yield and a larger mean NP size compared to water. Figure 5 displays TEM images of NPs obtained by laser ablation of Au, Ag, and Fe bulk targets in different solvents with 9-ns pulses at 1064 nm and 10 J cm^−2^ [166]. Moura and coworkers [165] hypothesized that, when acetone molecules are adsorbed around the metal NP, a protective surface dipole layer is developed in the most external plane, inducing a repulsive interaction between nanoparticles. NP aggregation in ethanol can be more intense, since ethanol is a low-polarity solvent compared to acetone. However, it was reported that ablation processes performed in ethanol environment had a low ablation efficiency [165]. This was attributed to the ethanol decomposition during ablation process, promoting the formation of permanent gas bubbles. The latter, in combination with the ablated plasma plume and the as-formed NPs, may act as obstacles within the laser path, thus reducing the energy reaching the target. Recently, Kalus et al. [167] studied the effect of persistent microbubbles on nanoparticle productivity in laser synthesis of colloids, finding that the highest productivity and monodisperse quality is achieved in liquids with the lowest viscosities.

**Figure 6:**
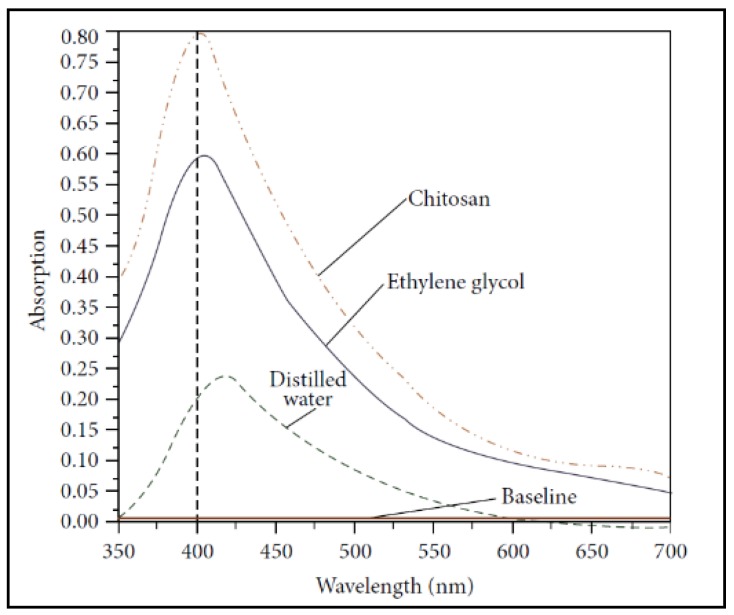
UV-visible absorption spectra of AgNPs prepared for 30 min ablation times in ethylene glycol, chitosan, and deionized water. Reprinted from [168], an open access article distributed under the Creative Commons Attribution License.

Tajdidzadeh et al. [168] showed that NPs ablation efficiency in chitosan solution is higher than in ethylene glycol (EG), and that it is higher in EG than in deionized water due to plasma confinement on the Ag target (Figure 6). It is worth noting that the broad tail at high wavelength values in AgNP UV-Vis spectra reported in the following is known to be ascribable to NP agglomeration [169]. Those who are not familiar with the UV-vis spectra of nanoparticles should refer to [22] for fundamental information on these phenomena.

In the same paper it is shown by TEM how chitosan solution, which has higher density and viscosity than other liquids, produces a mean size decrement for AgNPs. The same authors also assumed that the plasma generated on the target surface is confined, generating local high pressures and thus etching the target surface. The process, called secondary ablation, can improve ablation efficiency. Additionally, the obtained chitosan-functionalized NPs were shown to be quite stable because the biopolymer acts as a capping agent. 

Similar results was reported by Al-Azawi and coworkers [170], who synthetized AgNPs by laser ablation in three different solvents: water, ethanol, and polyvinylpyrrolidone (PVP). Indeed, the ablation efficiency for Ag colloids in ethanol was found to be the lowest, whereas in water it was higher than in PVP solution, corroborating the evidence that the ablation yield of AgNPs in organic solution is generally low. It was found that the efficiency of laser ablation increased, and the NP size decreased for the solvent with higher density and viscosity, which is in agreement with the findings of Moura and Tajdidzadeh.

Accordingly, also in Ganeev’s work [171] colloidal silver surface plasmon resonance (SPR) absorbance in water and in ethanol noticeably decreased over time as compared with AgNPs in ethylene glycol, which resulted more stable because of high solvent viscosity. This may be ascribed to a higher solvent viscosity, which prevents NP flocculation. 

Overall, we can state that the ablation efficiency is higher in an aqueous environment, but AgNPs are generally more stable in organic environment. This is correlated to solvent physicochemical properties, like the dipole moment and viscosity, which influence NP growth and stability. Higher solvent viscosity prevents NP flocculation and improves ablation efficiency and higher molecular dipolar moment of the solvent molecules generates a stronger electric double layer at the NP surface, which improves the repulsive forces between NPs, increasing their stability.

### 3.2. Pulse Duration

The effects of the pulse duration are dependent on the electron cooling time (electron-phonon coupling constant) of the material. For fs lasers, pulse duration is shorter than the electron cooling time; thus, the electron-lattice (phonon) coupling is negligible, and the ablation process can be considered as a solid–vapor transition. The ablation process associated with ns pulses is believed to be a thermal one, involving laser heating and melting [166,172]. For these reasons, during fs laser ablation, craters are clearer and more defined than during ns processes.

**Figure 7:**
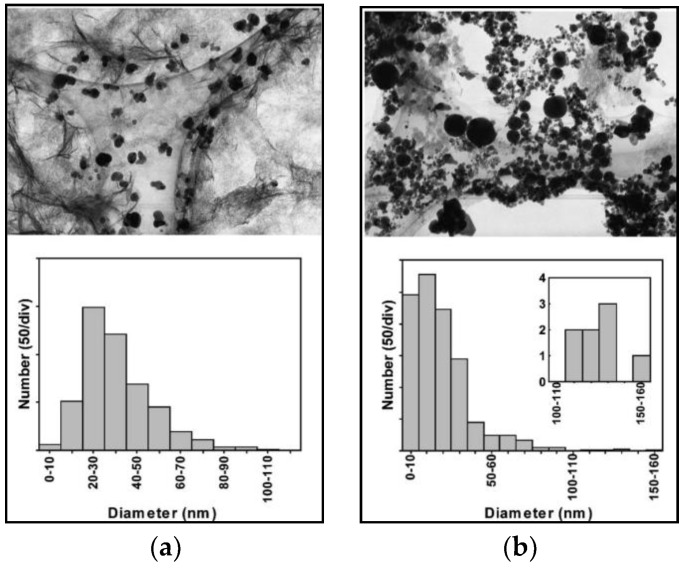
TEM images and size distribution of silver colloids prepared by (**a**) 120-fs and (**b**) 8-ns laser pulses. Reprinted from [173], with permission from Elsevier.

Tzuji et al. [173] reported a comparison between AgNPs ablated with ns and fs pulsed lasers. They showed that fs ablation yield was lower than nanosecond one. Sizes of ns-prepared particles were much dispersed, and they were irregularly shaped (Figure 7) as compared to fs-prepared particles. 

Barcikowski and colleagues [95] showed that fs pulses have an higher ablation rate than picosecond ones, but the reported process yield was about three times higher for ps laser ablation when compared to fs ablation. At the same time, it was shown that both ps and fs ultrashort pulsed lasers generated nanoparticles with comparable size distributions. A similar trend was found by Hamad [172], who reported a comparison between AgNPs produced by ns, ps, and fs pulses, and showed a higher ablation efficiency for fs pulses. 

### 3.3. Laser Wavelength 

According to the photon energy equation E = hc/λ, a shorter wavelength implies a greater energy. For instance, at a wavelength of 532 nm, green laser pulses have higher photon energy (2.33 eV) in comparison with those at a 1064-nm wavelength (1.16 eV). In general, the 532-nm wavelength is more effective at producing smaller AgNPs than the 1064-nm one. This is because the lower energy of 1064-nm photons results in less fragmentation, thus producing larger nanoparticles with a higher extinction coefficient in the near-infrared region. On the other hand, the fragmentation produced at 532 nm is higher not only because of the greater photon energy but also because this wavelength is in the range of the SPR peak of AgNPs, thus leading to a reduction of NP size in the colloidal solution [174].

The laser wavelength also determines the laser penetration into the metal target and, consequently, the ablation depth. This parameter decreases with the laser wavelength, thus indicating that the ablated mass per pulse may increase for longer wavelengths if reflectivity is the same [156]. 

**Figure 8:**
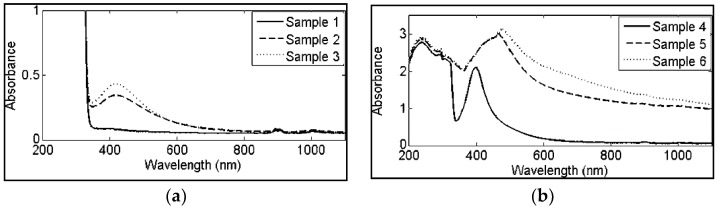
Absorption spectra of Ag nanoparticles in acetone prepared at (**a**) 532 nm wavelength; (**b**) 1064 nm wavelength at several fluences (samples 1 and 4: 14 J/cm^2^, samples 2 and 5: 18 14 J/cm^2^, samples 3 and 6: 22 J/cm^2^). Reprinted from [175], with permission from Springer Nature.

However, it is important to highlight that the influence of the laser wavelength on NP properties still depends on all the other laser parameters, e.g., pulse energy and duration, liquid media, and radiation focus, above all. Solati and colleagues [175] investigated the effect of laser wavelength on the production of AgNPs in acetone (Figure 8); they used nanosecond pulses at 532 nm and 1064 nm and, for each wavelength, they worked at different laser fluences. Results showed that NP size was smaller for a 532 nm than 1064 nm wavelength. They also showed that a surface plasmon resonance (SPR) shift between colloids produced at different fluences is more evident at 1064 nm than at 532 nm.

**Figure 9:**
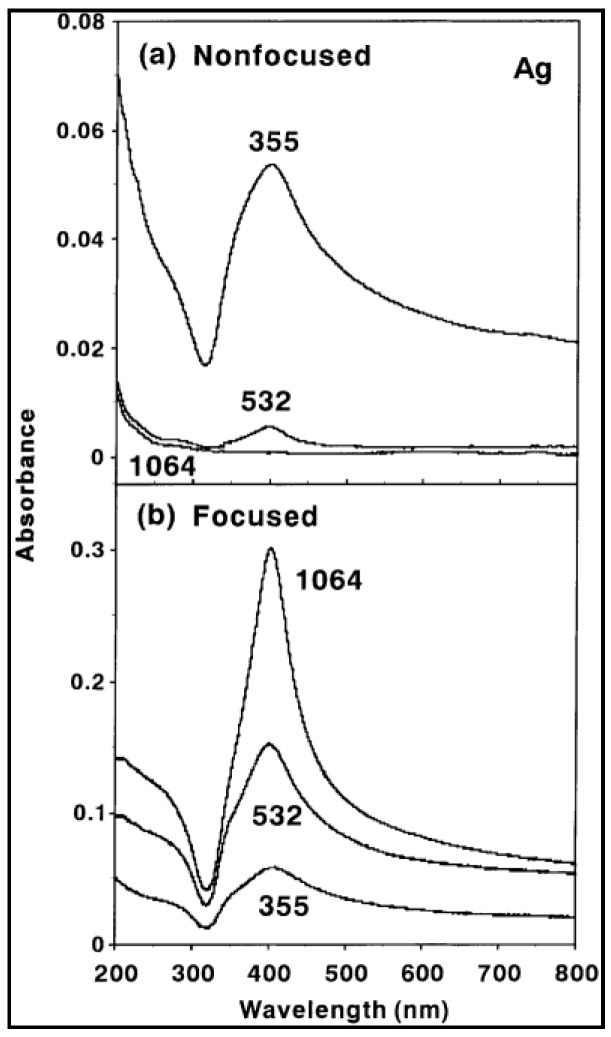
Absorption spectra of Ag colloidal solution prepared with various wavelength laser lights. (**a**) The laser beam was not focused and laser fluence was 900 mJ/cm^2^; (**b**) The laser beam was focused and laser fluence was >12 J/cm^2^. Reprinted from [176], with permission from Elsevier.

The studies by Tsuji and coworkers [176,177] showed that ablation efficiency (evaluated by measuring interband absorption at 250 nm) increases for shorter wavelengths when radiation is unfocused with respect to the target, while it increases with the wavelength for tighter beam focusing conditions (Figure 9). Authors hypothesized that ablation efficiency depends on the laser fluence, which changed with beam focusing. 

### 3.4. Laser Fluence and Energy Pulse 

Laser fluence is a crucial laser parameter that determines the ablation efficiency. NP production yield is affected by the cavitation bubble. In general, the cavitation bubble lifetime increases with the laser fluence [178]. As a result, when the time interval (determined by the repetition rate of pulsed lasers) between two subsequent pulses spatially overlapping on the same spot onto the target is faster than the bubble lifetime, the latter substantially shields and reflects the incoming pulse, thus reducing the ablation rate [156]. 

**Figure 10:**
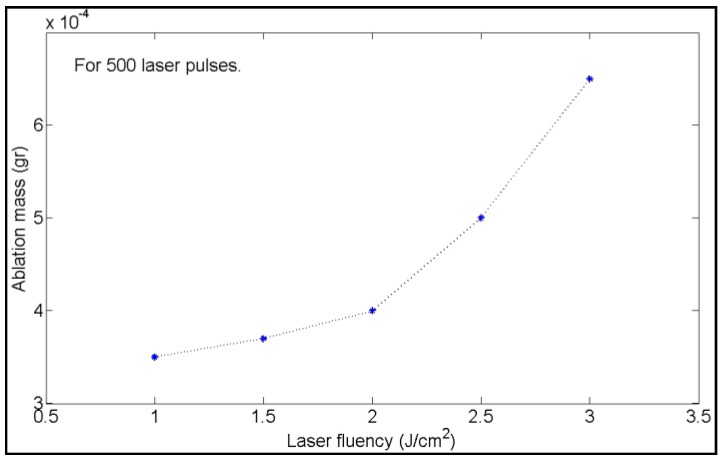
Ablated mass versus laser pulse fluency. Reprinted from [179], an open access article distributed under the Creative Commons Attribution License.

Above the ablation threshold, increasing the laser fluence gradually increases the synthesis yield. In fact, Moura et al. [165] observed that the absorption intensity tended to be higher at increasing laser fluences, suggesting that AgNPs concentration increased. Dorranian and coworkers [179], for example, showed the increase of ablated mass upon increasing the fluence (Figure 10).

**Figure 11:**
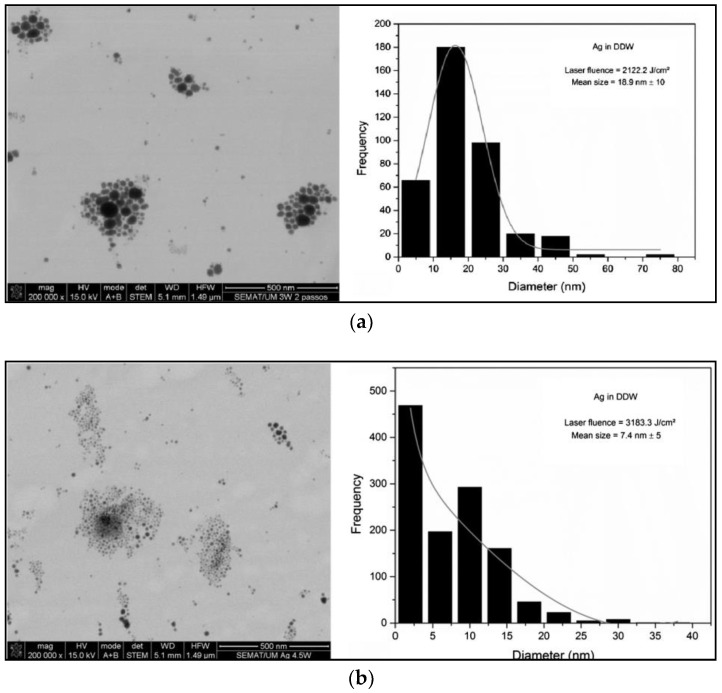

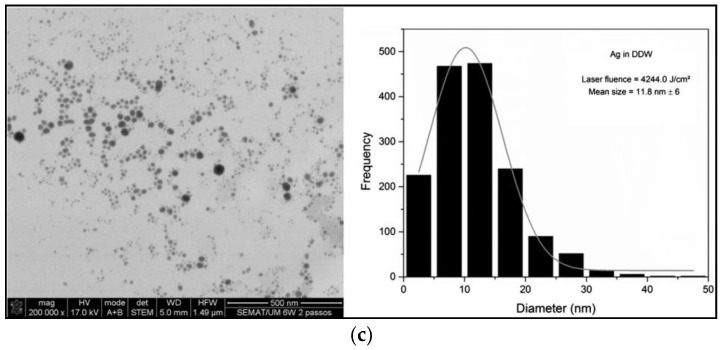
STEM images and corresponding histograms of silver colloidal nanoparticles prepared by laser ablation at different laser fluences (**a**) 2122.2 J/cm^2^; (**b**) 3183.3 J/cm^2^ and (**c**) 4244.0 J/cm^2^. Reprinted from [165], with permission from Elsevier.

They also found that smaller average NP sizes were obtained by using higher laser fluence values, as shown in STEM (scanning transmission electron microscope) micrographs of Figure 11. This behavior was explained considering that, at high pulse energies, the ablation process is accompanied by melting of the target surface, with less evaporation and NP auto-absorption of laser light. This absorption leads to the formation of smaller particles as a result of fragmentation of larger ones.

This trend was also found in other papers [175,179,180,181]. However, Nikolov et al. [182] demonstrated that the average particle size remained unchanged with the laser fluence at its fundamental wavelength (λ = 1064 nm), but it increased strongly with increasing laser fluence at the second harmonic wavelength (λ = 532 nm). On the contrary, Al-Azawi [170] found that the ablation efficiency increased (while the particle size decreased) when laser fluence reached its maximum; subsequently, the ablation efficiency rapidly decreased with increasing the laser fluence. This behavior was ascribed to the occurrence of auto-absorption processes leading to fragmentation of larger particles. 

Ablation efficiency generally increases with increasing of pulse energy, as was shown by Valverde-Alva et al. [183] who found an increase of the SPR peak intensity when higher laser pulse energies were used. This result was attributed to the more concentrated AgNP colloids produced with higher pulse energies. 

Syntheses of AgNPs using a 1064-nm wavelength Nd:YAG laser, with pulse frequency of 20 Hz and 4 ns of pulse duration, were also carried out by our group. We observed that, at a fixed ablation time of 20′, ablation rate increased with pulse energy. Moreover, AgNPs average size decreased with the energy. Moreover, we found that ablation rate grew with pulse energy for longer ablation times. This effect was attributed to the increasing concentration of AgNPs lying on the laser beam path, which caused an attenuation of the incident radiation owing to scattering phenomena.

### 3.5. Repetition Rate 

The repetition rate (RR) is defined as the number of output laser pulses per unit time. Therefore, for a given laser power, reducing the repetition rate results in an increase of the pulse energy, thus yielding a higher ablation rate per pulse (and larger cavitation bubbles) because of the increased laser fluence [156]. In general, ablation efficiency and concentration of AgNPs increases with the repetition rate. To explain this phenomenon, Valverde-Alva measured the transmission of laser pulses through colloidal solutions and showed that it increased with the RR. Zamiri and coworkers [184] obtained a similar trend. They also investigated the variation of average AgNP size and showed that it increased with increasing RR, in contrast to the trends obtained by Menéndez-Manjón and Barcikowski [185]. However, in this last case, significantly higher repetition rates, spanning in a broad range from 100 to 5000 Hz, were used compared to the very limited range of RR from 10 to 40 Hz explored by Zamiri. For RR exceeding tens of kHz or even approaching the MHz regime, the pulse energy must be decreased to reduce the size and lifetime of the cavitation bubble, which otherwise would shield the laser radiation, thus reducing the ablation yield [97].

## 4. AgNPs in Food Packaging 

AgNPs are a valid antimicrobial additive to control the microbial population in commercialized foodstuff. They have been proved to reduce, delay, or inhibit the growth of spoilage microorganisms, thus improving food shelf life. 

Some recent reviews and book chapters discuss the use of AgNPs for this specific application [8,20,21,23,24,25,26,27,28,29,30,31,186,187]. These references highlight the antimicrobial activity of AgNPs, the mechanism of action against microorganisms, and the correlations existing between bioactivity and AgNP features such as size, shape, and concentration. In most of the literature in this field, AgNPs are embedded in packaging films and tested against foodborne microorganisms, or directly used to package fruit and vegetables, meat, and dairy products. For example, Costa and coworkers [188] prepared and used silver-montmorillonite (Ag-MMT) in packaging for freshly-sliced fruit salads, and found inhibition of microbial growth and increased shelf life. In another interesting work, Sivakumar and colleagues [189] showed a new method to produce nontoxic silver nanorods from dairy industry waste. They used them to control bacterial growth in milk during processing and storage, thus demonstrating extension of milk shelf-life. The most cited reviews and book chapters include many other examples of real-life use of AgNPs. Table 3 summarizes some of the aforementioned applications of AgNPs in food packaging.

The antimicrobial properties of packaging materials are based on the migration of antimicrobial substances from the packaging to the food, and/or to the headspace surrounding the food product. Thus, the migration of an active compound from the substrate is an intentional process, which is needed to exert the antimicrobial and protective action against undesirable food contaminants [194]. For these reasons, the efficiency of antimicrobial packaging is largely determined by a controlled release of the antimicrobial agent from the active material. A slow and gradual migration of these substances allows for maintaining an effective antimicrobial concentration on the product over time [194,195]. Nerin and colleagues [194] highlighted that antimicrobials needed to reach bacterial cells to exert their action, while other substances such as antioxidants can exert their action even without being in direct contact with foodstuffs and without releasing any agents. It is worth pointing out that other toxic substances can be released unintentionally into food. AgNP safety limits, both for the environment and human health, are different according to the various legislative authorities in Europe, the USA, Japan, and Australia [196]. For instance, in the United States and in Japan, only silver nitrate is regulated by law in the food and drinks sector, with a maximum allowed limit of 0.017 mg/kg for foodstuffs and 0.1 mg/kg for drinking water, respectively. As for nanosilver, colloidal solutions are accepted in the United States and commercialized as nutrition supplements (e.g., Mesosilver), with the claim of being highly beneficial for human health. In the medical field, different wound dressings containing nanocrystalline silver or silver ion-releasing systems are widely spread as well as indwelling devices, like prostheses or catheters [196]. To date, the European Union does not recommend silver for medicinal use, because of the lack of reliable information with respect to health-risk assessment. The European Food Safety Authority (EFSA) restricted food migration to a maximum of 0.05 mg/kg [196,197]. 

Besides silver ions, excessive release of entire silver nanoparticles from packaging can be dangerous to human health. Food and beverages produced with AgNPs added in their packaging are considered as the main source of exposure to nanoparticles through ingestion [198]. After ingestion, these nanoparticles undergo various chemical reactions, including agglomeration, adsorption, or binding with other food components, and reactions with acids and digestive enzymes. Internal systemic exposure to NPs can be hazardous since these particles are able to cross biological barriers and reach internal body tissues [154]. As a consequence, AgNPs may accumulate in tissues, resulting in changes in body nutrient profiles. In additions, nanoparticles may introduce toxic agents or viruses adsorbed on their surfaces, or induce production of oxyradicals at the cellular level [198,199]. Therefore, regulation is very important to minimize harmful consequences deriving from the use of nanoparticles, although there are still no internationally recognized research protocols or standards [198].

## 5. Conclusions

In this review, we focused on AgNPs, a very useful nanomaterial for active food packaging, paying particular attention to AgNPs synthetized by LASiS. After brief overviews of bioactivity pathways of AgNPs and of synthesis methods to produce silver nanocolloids, we focused on the LASiS method, describing in detail its working principles and the influence of the main laser parameters on the production yield and quality. A short overview of the use of AgNPs in food packaging was also proposed. Laser ablation is a green technique to produce stable Ag nanocolloids in a wide variety of dispersing media without using metal precursors and reductants. Highly pure colloids are produced with unique surface characteristics and without any by-products. These features, in principle, make AgNPs produced by LASiS some of the best candidates for antimicrobial applications. However, to date, the productivity is insufficient for direct use in the industrial sector. The current world record for LASiS NP productivity is 4 g/h; this value, although appealing, needs to be improved for LASiS to be used in the industrial sector. Methods to increase this productivity level are currently under development, exploiting high scanning speeds. This way, laser-induced cavitation bubbles are spatially bypassed at high repetition rates, and continuous multi-gram ablation rates have been already demonstrated for platinum, gold, silver, aluminum, copper, and titanium. LASiS requires a great amount of energy, and its scaling-up at the industrial production level is approaching the efficiency required for real-life applications, although there is still a need of for further technological improvement, mostly with regard to the technological laser solutions. 

## Figures and Tables

**Table 1:** Activity of Ag^+^ and/or AgNPs against selected bacterial strains. Adapted from [124], with permission from John Wiley and Sons.

Target Organism	Different Form of Silver	References
*Staphylococcus aureus* and *Escherichia coli*	Silver ions	[125]
*Escherichiacoli*	Silver nanoparticles	[126,127]
RNA viruses	Silver ions	[128]
*Escherichia coli*, *Vibrio cholerae*, *Pseudomonas aeruginosa*, and *Salmonella typhus*	Silver nanoparticles	[129]
*Escherichia coli*	Silver ions	[130]
*Escherichia coli*, *Salmonella typhi*, *Staphylococcus epidermidis*, *StreptococcusAureus*	Silver nanoparticles	[131]
*Phoma glomerata*, *Phoma herbarum*, *Fusarium semitectum*, *Trichoderma specie* and *Candida albicans*	Silver nanoparticles	[132]
*Escherichia coli*, *Streptococcusaureus,* and *Pseudomonas aeruginosa*	Silver nanoparticles	[133]
*P. aeruginosa*, *S. aureus*, pathogenic fungi *Aspergillus flavus* and *Aspergillus niger*	Silver nanoparticles	[134]
*S. aureus*, *E. coli*, *Klebsiella pneumoniae*, *Bacillussubtilis*, *Enterococcus faecalis*, *P. aeruginosa*	Silver nanoparticles	[135]

**Table 2:** Effects of Ag-NPs at different ranges of concentration on different cell lines. Adapted from [52], with permission from Elsevier.

Concentration Range	Effects of AgNPs	References
25–75 μg/mL	In rat alveolar macrophage cell line, cytotoxicity increases in a concentration-dependent manner	[141]
5, 15, 40, 125 μg/mL	Cytotoxicity occurred through mitochondrial depolarization	[142]
20–250 μg/mL	Apoptosis and necrosis induced in an Hematopoietic stem cell (HSC) cell line	[143]
1, 2, 4 μg/mL	Cell viability decreased in a concentration-dependent manner	[144]
0.4 and 0.8 μg/mL	Arrest G1 phase in cell cycle in a Murine Macrophages cell line (#RAW 264.7)	[145]

**Table 3:** Applications of AgNPs in food industry. Adapted from [25], with permission from Elsevier.

Nanomaterial Product	Packaging Manufacturer	Country	NP Size	References
Nano-silver salad bowl	Changmin Chemicals	Korea	not reported	[190]
Nano silver baby mug cup and nurser	Baby Dream^®^ Co., Ltd.	Korea	not reported	[191]
Fresh Box^®^ food storage containers	BlueMoonGoods^™^	USA	not reported	[190]
FresherLonger^™^ containers and bags	SharperImage^®^	USA	25 nm and 1–100 nm	[192]
Nano-silver storage box	Quan Zhou Hu Zeng Nano Technology^®^ Co., Ltd.	China	not reported	[190]
Plastic food containers and water bottle	A-Do Global	Korea	not reported	[191]
Fresh food containers	Oso Fresh	USA	40–60 nm	[193]
Smartwist food storage with nano-silver	Kinetic Go Green	USA	10–20 nm	[193]
